# Weight management communications in idiopathic intracranial hypertension: challenges and recommendations from the patients’ perspective

**DOI:** 10.1136/bmjno-2023-000527

**Published:** 2023-12-09

**Authors:** Sally Abbott, Amanda Denton, Sui H Wong, Susan P Mollan, Kim CM Bul

**Affiliations:** 1 Institute for Health and Wellbeing, Centre for Healthcare and Communities, Coventry University, Coventry, UK; 2 Department of Dietetics, University Hospitals Coventry and Warwickshire NHS Trust, Coventry, UK; 3 IIH UK Registered Charity, Washington, UK; 4 Department of Neuro-Ophthalmology, Moorfields Eye Hospital NHS Foundation Trust, London, UK; 5 Medical Eye Unit, Guy's and St Thomas' Hospitals NHS Trust, London, UK; 6 Faculty of Life Sciences and Medicine, King’s College London, London, UK; 7 Institute of Neurology, Department of Clinical and Movement Neuroscience, University College London, London, UK; 8 Birmingham Neuro-Ophthalmology, University Hospitals Birmingham NHS Foundation Trust, Birmingham, UK; 9 Institute of Metabolism and Systems Research, Translational Brain Science, University of Birmingham, Birmingham, UK; 10 Institute for Health and Wellbeing, Centre for Intelligent Healthcare, Coventry University, Coventry, UK; 11 Renal Services, University Hospitals Coventry and Warwickshire NHS Trust, Coventry, UK

**Keywords:** CLINICAL NEUROLOGY, HEALTH POLICY & PRACTICE, METABOLIC DISEASE, OPHTHALMOLOGY

## Abstract

**Background:**

Idiopathic intracranial hypertension (IIH) is a neurometabolic condition severely impacting the quality of life of people living with IIH (PwIIH). Most PwIIH are overweight or live with obesity, and weight loss is recommended by healthcare professionals (HCPs) as it is central to disease management. There is currently no research evaluating patient–clinician interactions when discussing weight management in IIH. The aim of this study was to evaluate the patient experience of communication with HCPs regarding weight management from the perspective of PwIIH.

**Methods:**

A cross-sectional online survey was developed and distributed by the IIH UK charity via their mailing list and social media network. Eligible participants were adults with IIH who have been recommended to lose weight by their HCP. Descriptive statistics were used to summarise quantitative responses and content analysis was used to inductively draw out themes from open-ended free-text responses.

**Results:**

There were 625 respondents. One-fifth of PwIIH (n=127/603, 21%) felt that HCPs were supportive and empathetic about weight management. Five themes were identified on how experiences regarding weight management for IIH can be improved, with PwIIH recommending for HCPs to: (1) detail the relationship between IIH and weight, (2) individualise care, (3) give advice, (4) provide support and (5) adapt communication.

**Conclusion:**

The majority of PwIIH recalled a poor experience and negative emotions when engaged in discussions regarding weight management with their HCPs. Further research should explore the HCPs perspective and evaluate interventions aiming to improve the quality of patient–HCPs communication in IIH.

**WHAT IS ALREADY KNOWN ON THIS TOPIC**

- Discussions about weight management are a sensitive topic for patients and evidence shows that healthcare professionals (HCPs) lack confidence in discussing body weight with patients. Priority setting research by the James Lind Alliance highlighted that weight management in the context of idiopathic intracranial hypertension (IIH) is a top research priority for patients and HCPs. To date, there is no published research evaluating the experiences of weight management interactions from the perspective of people living with IIH (PwIIH).

**WHAT THIS STUDY ADDS**

- This is the first study evaluating the experiences of weight management interactions with HCPs from the perspective of PwIIH. The results of this study demonstrate that PwIIH feel unsupported and experience negative emotions when weight management discussions are initiated by their HCPs. This study also provides unique insights into how HCPs could improve their approach to weight management; by explaining the relationship between IIH and weight, providing personalised care, giving tailored advice, providing support and adapting communication style and the setting of communication.

**HOW THIS STUDY MIGHT AFFECT RESEARCH, PRACTICE OR POLICY**

- The results of this study provide for the first time the experience of PwIIH and their recollection of weight management discussions with HCPs and they offer suggestions that would make the interactions more effective. This research highlights the need for specific training for HCPs in the context of weight management for IIH to support improved healthcare accessibility, promote non-stigmatising practices and improve both patient and clinician satisfaction in this context.

## Introduction

Idiopathic intracranial hypertension (IIH) is characterised by increased intracranial pressure leading to reported signs and symptoms such as headache, pulsatile tinnitus and papilloedema, with the potential risk of permanent visual loss.^1 2^ Although the exact causes of this condition remain unknown it occurs more often in women of childbearing age compared with men and children as well as in people who are overweight (body mass index, BMI, 25–30 kg/m^2^) or who live with obesity (BMI≥30 kg/m^2^).^3^ The global annual IIH incidence rates range from 0.03 to 2.36 per 100 000 and are positively associated with country-specific obesity rates.^4^ In the UK, there has been a stepwise increase in the incidence and prevalence of IIH with increasing numbers of people being admitted to hospital care.^1 5–7^

The use of specialised healthcare by people living with IIH (PwIIH) causes a significant economic burden on the National Health Services (NHS) with estimated direct healthcare resource use costs of £462 million per annum by 2030.^5^ This has also been demonstrated in the USA.^8^ Additionally, research states that PwIIH experience low quality of life mainly due to their headache symptoms^9^; however, research into other factors contributing to this is limited. This stresses the importance of more research into effective and acceptable weight management interventions for PwIIH.

There is a striking association with increased BMI and moderate weight gain.^1 4 6 10^ The disease is modified by weight loss and a recent randomised controlled trial evaluating bariatric surgery and a multicomponent lifestyle intervention found that weight loss mirrored reduction in intracranial pressure.^11 12^ A weight loss in the region of between 3% and 24% has been shown to reduce symptoms and lead to remission of the disease whereas regaining 6% of body weight has been shown to be associated with recurrence of IIH among some PwIIH as demonstrated by one study.^2 12 13^ The condition is managed by neurologists, ophthalmologists, neurosurgeons and more recently interventional radiologists, none of whom have formal training in weight management.^14^

A recent qualitative systematic literature review highlighted that weight stigma within patient–healthcare professionals (HCPs) interactions has a negative impact on healthcare access and quality of healthcare provision from the perspective of people living with obesity.^15^ People who have obesity perceive there to be negativity around their weight status based on the language used by HCPs and often feel ashamed, humiliated and blame themselves for having obesity. At the same time, due to a lack of knowledge base, skill set and guidelines, HCPs experience many challenges when communicating about weight with people who have obesity. There is a clear unmet clinical need for education and specialised training among HCPs on how to communicate about body weight change and weight status. This is important as HCP interactions about weight management can negatively affect future weight trajectory and the dialogue with people who have obesity.^16^ Although HCPs specialising in neurology are medically trained to diagnose PwIIH, they are not likely to have received training in obesity management. This could potentially lead to ineffective consultations where the importance of sustained weight reduction is not addressed or managed effectively.

Patient and public involvement and engagement in research and healthcare practices is becoming increasingly important to generate meaningful healthcare impact.^17^ This has been reflected in a recent research study in which a priority setting exercise was performed with PwIIH and HCPs in accordance with the James Lind Alliance Priority Setting Partnership.^18^ There is limited evidence in IIH where patients are central to the conduct of the research, and a previous study has shown the benefit of this close working relationship.^19^ Research into the role of weight management in PwIIH was highlighted as a research priority, including approaches that provide sensitivity in discussing weight and address the stigma associated with obesity.^2 18^ Despite this, there are currently no published research studies on the experiences of PwIIH when HCPs communicate about the role of body weight and weight management in IIH. Hence, the aim of this study was to evaluate the experiences of PwIIH in their interactions with HCPs regarding weight management.

## Methods

### Study design and participants

A cross-sectional study was undertaken and collected data using the SurveyMonkey platform. The survey was open to responses between 26 April 2021 and 14 May 2021. Eligible participants were people with a diagnosis of IIH, aged over the age of 18 years old, and who had been advised to lose weight by any HCP.

### Survey development

The questionnaire was developed in response from patients citing concerns around the patient–HCPs weight management interactions as expressed by PwIIH across diverse social media groups. A steering committee of Trustees of the charity IIH UK, which included two PwIIH, led on the questionnaire development. The questions within the survey were formulated based on patient experiences voiced to the charity IIH UK by PwIIH, primarily via Facebook and Twitter social media channels. The survey was piloted with three PwIIH which led to minor modifications to the questionnaire wording to aid the use of plain English language readability (see online supplemental file 1).

10.1136/bmjno-2023-000527.supp1 Supplementary data

### Data collection

The survey was distributed to 395 members of the charity IIH UK via their membership mailing list. The survey was also distributed via social media channels, including the ‘IIH UK National Charity’ Facebook page and the @IIHUK Twitter handle. All responses were anonymous, and questions permitted both closed and open responses from participants, giving participants the opportunity to expand on their given answer in their own words. Participants were able to answer all the questions however no single question was mandated to be filled out. Additionally, the participants could select one or more options at certain questions. IP addresses for each response were cross-checked to ensure that responses were submitted only by PwIIH residing in the UK.

### Data analysis

Responses to closed questions were analysed using frequency descriptive statistics (n, %). Open free-text responses were analysed using content analysis. Content analysis provides a subjective interpretation of text data through a systematic process of coding and categorising data into themes^20^ and is a convenient method used in health research to analyse textual types of data, including open-ended survey questions.^21^ A coding frame was developed using an inductive approach to determine themes from the data. Quotation excerpts were extracted and presented to illustrate findings and enhance credibility.

## Results

**Figure 1:**
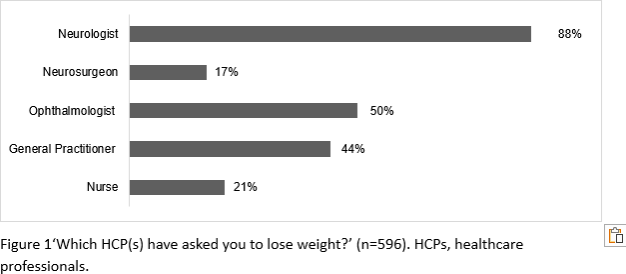
‘Which HCP(s) have asked you to lose weight?’ (n=596). HCPs, healthcare professionals.

A total of 625 PwIIH completed the survey. IIH UK charity has a wide national reach through its mailing list and social media network but exact numbers on how many people were exposed to the online survey is unknown. The most frequently reported HCP advising PwIIH to lose weight was a neurologist (526/596, 88%), an ophthalmologist (297/596, 50%), a general practitioner (267/596, 44%), a nurse (129/596, 21%) and a neurosurgeon (100/596, 17%) (figure 1).

### Weight management interactions

**Figure 2:**
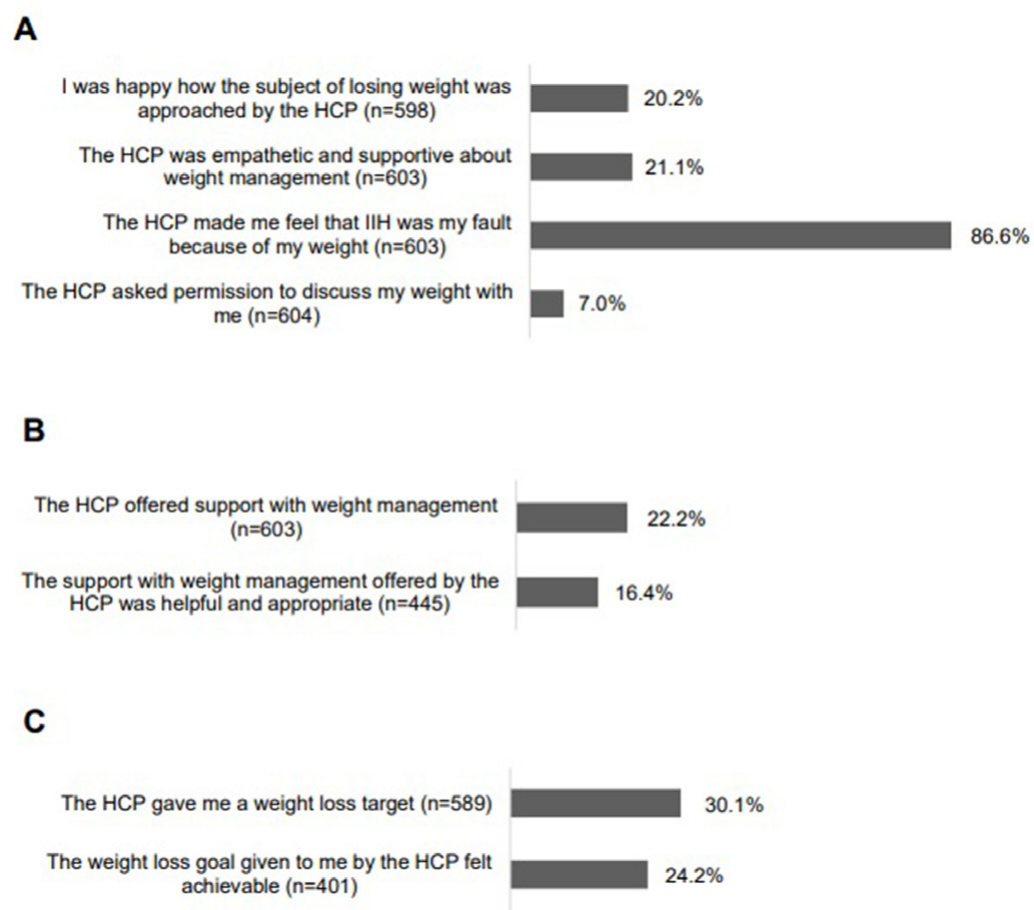
(A) experiences of PwIIH HCP raising the topic of weight, (B) experiences of PwIIH HCP support with weight management, (C) experiences of PwIIH HCP setting weight loss targets. HCP, healthcare professional; PwIIH, people living with idiopathic intracranial hypertension.

**Figure 3:**
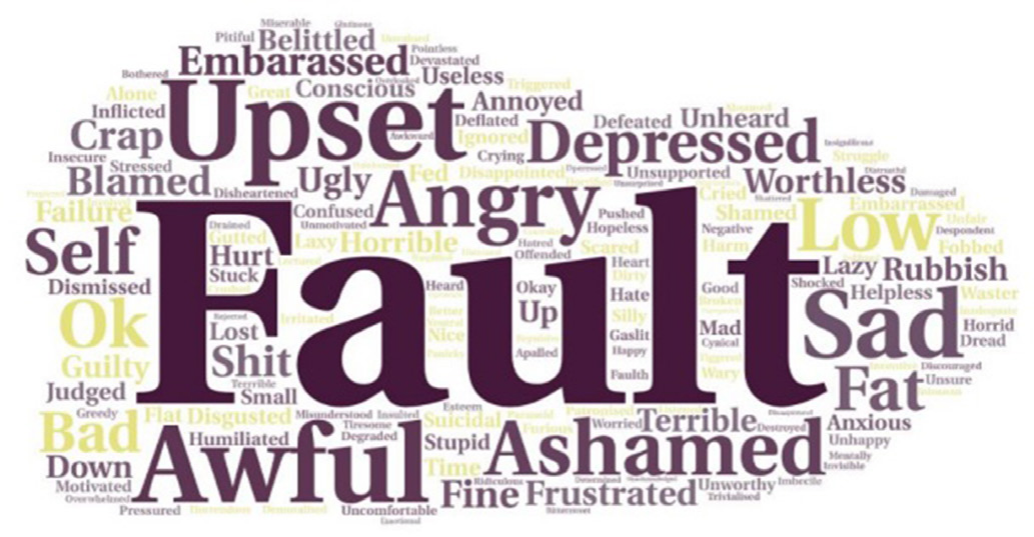
Word cloud analysis of responses to question ‘How did being advised to lose weight make you feel?’ (n=602).

Most PwIIH (522/603, 87%) recalled that the HCP made them feel that IIH was their ‘fault’ due to their weight. Only 7% (42/604) of PwIIH reported being asked by the HCP for permission to discuss their weight. One-fifth (121/598, 20%) of PwIIH were ‘happy’ with how their weight was discussed with them and 21% (127/603) of PwIIH felt that the HCP was empathetic and supportive about weight management (figure 2). When PwIIH were asked how being told to lose weight made them feel, the ten most frequent responses were negative emotions: ‘fault’ (n=80), ‘upset’ (n=42), ‘embarrassed’ (n=37), ‘awful’ (n=37), ‘ashamed’ (n=35), ‘angry’ (n=32) ‘sad’ (n=30), ‘depressed’ (n=28), ‘blamed’ (n=24), ‘worthless’ (n=23) and ‘fat’ (n=19) (figure 3).

### Weight management support

**Figure 4:**
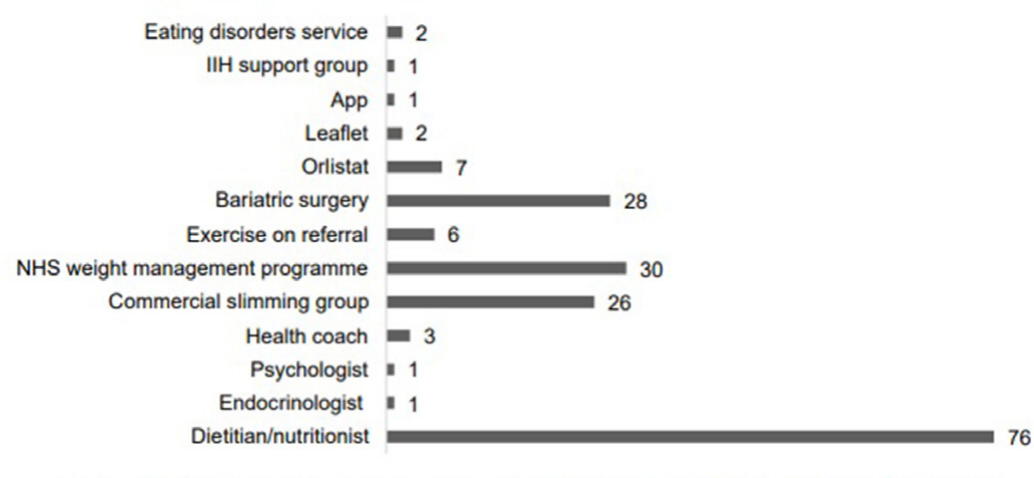
Weight management support offered to PwIIH by an HCP (n=462). HCP, healthcare professional; PwIIH, IIH, idiopathic intracranial hypertension; PwIIH, people living with idiopathic intracranial hypertension.

Only 22% (134/603) of PwIIH reported that the HCP offered weight management support and 16% (73/445) felt that this support was ‘helpful and appropriate’. HCPs offered a variety of weight management interventions (figure 4), with referral to a dietitian/nutritionist (n=76) offered most frequently, followed by an NHS-funded weight management programme (n=30), referral for bariatric surgery (n=28) and a commercial slimming group (n=26).

### Weight loss targets

HCPs provided weight loss targets to less than a third (177/589, 30%) of PwIIH. Less than a quarter (97/401, 24%) of PwIIH felt the weight loss target was ‘realistic’ (figure 2). Table 1 shows participant quotation excerpts illustrating the weight loss targets advised by their HCP and whether they felt the target was realistic or not.

**Table 1:** Quotation excerpts from unique participants recalling weight loss targets set by the HCP and whether they did or did not feel this was realistic

Realistic weight loss goals	Unrealistic weight loss goals
‘Was asked to lose 5% and achieved 7%.’	‘It sounded like a big number. 13% of your body weight sounds huge.’
‘The doctors told me to create my own goals and they should not be super goals or unachievable in a short period. So I am managing smaller goals one at a time.’	‘It was the lowest end of healthy BMI and it just seemed impossible that my body would ever be that small.’
‘Approx 1 kg per week of steady loss. Calorie reduction of approx 100–200 per day.’	‘I was told to lose at least 10% of my body weight within a few months. No support or help was given to help me achieve that.’
‘Between 5% and 10% of my body weight in a year.’	‘10st is just so huge, it feels like a mountain I can’t climb.’
‘I was told 10% of my original weight was often enough to see improvement. In reality, I had to lose a lot more than that but 10% was quite easy to achieve.’	‘Yes my initial goal was 10% which I achieved but it made no difference to symptoms so I was told to keep going until it works. Now no goal and no end in sight.’
	‘They kept moving it… 5%, 6%–10% 12%, then I didn't lose it fast enough.’
	‘Got told to lose 15 stone I am no good with massive goals like that.’
	‘He just looked at me and said I needed to lose a lot of weight. Wasn’t even weighed.’
	‘The goal I was given was not realistic as it is what I weighed 20 years ago when I was a 15 year old adolescent.’
	‘I was told 10% of my body weight. I have lost quite a bit more than that, but yet my doctor’s still insist if I lost more weight my symptoms would improve.’

BMI, body mass index; HCP, healthcare professional.

### Improving the weight management patient experience

Online supplemental file 2 outlines the 5 categories and 18 subcategories that were derived from free-text responses to questions about how the experience of treating IIH via weight management could be improved from the perspective of PwIIH, alongside illustrative quotations.

10.1136/bmjno-2023-000527.supp2 Supplementary data

#### The relationship between IIH and weight

PwIIH wished that HCPs explore the relationship between IIH and excess weight and to ‘provide an explanation’ of the biological mechanisms of excess weight that may cause or exacerbate symptoms of IIH; but to also ‘acknowledge weight as a risk factor, not a cause’ of IIH. PwIIH referred to the condition itself being named idiopathic (meaning of ‘unknown cause’), thus perceiving that IIH cannot be caused by excess weight alone. And they reasoned that not all PwIIH have excess weight and that comparatively very few women of childbearing age with obesity in the population have IIH, either. There was a sense of scepticism among PwIIH around the relationship between IIH and excess weight and, therefore, wanted HCPs to ‘Provide evidence’ and ‘actual scientific proof’ to them.

#### Individualising care

PwIIH felt that weight management discussions should be more individualised. HCPs could ‘consider the impact of IIH symptoms’ when providing any lifestyle advice for weight management; citing that symptoms of pain and fatigue were a challenge to adopting helpful physical activity and eating behaviours. Some PwIIH expressed that HCPs should ‘investigate comorbidities’ aside from IIH, as barriers to their weight management. PwIIH also felt that HCPs should ‘explore mental health’ since they felt that the symptoms of IIH contributed to mental illness and vice versa that mental illness presented challenges for their weight management. To provide individualised care, PwIIH expressed that HCPs should ‘get to know the ‘person’ without making assumptions and expressed that they did not want to be ‘treated like a number’.

#### Giving advice

When being given advice on weight management, PwIIH felt that HCPs should acknowledge that IIH is ‘complex’ to self-manage and that HCPs should ‘avoid oversimplistic advice of ‘eat less, move more’. PwIIH wanted HCPs to ‘provide realistic weight loss targets’ which should be quantified both in terms of amount of weight loss and specified time frame.

#### Providing support

PwIIH wished for improved weight management support to be provided by HCPs. PwIIH wanted HCPs to ‘discuss the available options’ with them, which would contribute to PwIIH feeling more ‘in control’ of their care and solutions were being offered. PwIIH did not expect that the HCP managing their IIH should be expected to provide isolated weight management support and wanted their HCP to ‘organise referrals to other HCPs’, in particular to weight management services or HCPs trained in lifestyle management (eg, dietitians, exercise physiologists). PwIIH expressed that HCPs could also ‘provide resources for self-management’ in the form of leaflets which offered ‘practical’ support.

#### Communication

PwIIH felt that HCPs could adapt their communication style and approach when discussing weight management. PwIIH felt that HCPs should ‘ask for permission’ before discussing or measuring weight. PwIIH felt HCPs should ‘Show empathy’ and alter their choice of language when discussing weight management; including ‘use of people-first language’ not using terms such as ‘obese’ and ‘avoid using shaming language’ which could result in PwIIH feeling‘blamed’ for their illness. PwIIH wished for HCPs to ‘engage in a two-way dialogue’ about their excess weight as part of shared decision-making, rather than being ‘talked at’. The setting in which weight management conversations took place was important to PwIIH. They expressed that HCPs should ‘ensure privacy’ and not have weight management discussions where privacy could not be maintained, such as in inpatient settings, or vocalising in waiting rooms what investigation they were being called in for that is, to be weighed.

## Discussion

This study provides novel insights into the experiences of PwIIH regarding their current, mostly negative, interactions with HCPs about body weight and weight management. It offers suggestions from PwIIH on how the communication of weight management could be improved in the context of IIH disease management. Despite significant evidence demonstrating that weight loss is an effective treatment for IIH^10–12^ we found that only a fifth of PwIIH recalled being offered support for weight management and only 16% of PwIIH found the support to be ‘helpful and appropriate’. This evidence demonstrates an unmet clinical need to provide training and support in regard to approaching weight management conversations and onward referrals to weight management service, where appropriate, for HCPs who care for PwIIH.

PwIIH expressed that it was important to them that the HCP should first ask their permission to discuss weight. However, less than 1 in 10 (7%) of PwIIH in our study recalled being asked for permission first. Asking questions, rather than issuing statements, is a core principle of motivational interviewing which has been shown to facilitate patient-driven behaviour change^22^ and is recommended in fields such as pain neuroscience to enhance a patient’s receptiveness to advice and education.^23^

The way in which HCPs communicated about weight management invoked a negative emotive response among PwIIH, with most reporting feelings of self-blame and ‘fault’ for having IIH due to their weight. Research has shown that providing a biological explanation for disease processes is effective at reducing disease-related stigma^24^ and shifting the focus from personal blame to physiological mechanisms can be an effective means to avoid using stigmatising language and inciting blame.^25^

Setting targets and goals is considered an important behavioural change technique in weight management. In our study, PwIIH expressed that they found weight targets to be helpful for their weight management; however, most PwIIH recounted that the targets set were unrealistic to attain. Providing targets for weight loss in IIH are yet to be determined, as while most studies recommend a level at which the disease remits, none have yet to assess this as their primary aim. Unrealistic weight loss targets are cited in the literature as a barrier to weight management in the general obesity population,^26^ with the ‘false hope’ hypothesis suggesting that very ambitious targets are less likely to be met and leads to disappointment, dissatisfaction and decreased effort.^27^ On the contrary, systematic review data have found that realistic and specific goal setting is effective for weight management among people with obesity.^28^

Our findings also provide suggestions to improve clinical communication from the perspective of PwIIH. More effective weight management encounters as part of treatment for IIH could be enabled by HCPs explaining the evidence of the relationship between IIH and weight, individualising care, giving advice, providing support and adapting communication. This could potentially contribute to better treatment adherence and improve self-management practices as seen in chronic conditions, including obesity.^29 30^

Our study has several strengths. Embedding active patient and public involvement ensured the research was relevant to service users and user-friendly survey question.^19 31^ Although it is unknown how many PwIIH have been exposed to the online survey, the method of survey distribution via IIH UK charity platforms meant that a large UK sample of PwIIH completed the survey. However, this study also has some limitations. Due to the nature of the survey distribution, it was not possible to calculate a response rate. For future occurrences, the online survey could be posted on IIH UK charity webpage so the number of views and responses can be tracked to calculate a response rate. Although this was an anonymous survey which may have contributed to a higher response rate, there were no data on participant characteristics and therefore it is not certain how representative the study sample is of the target population. As the survey was distributed online, people without access to the internet and people with lower levels of digital literacy are likely not have been included.^32^ As the responses were collected retrospectively of healthcare encounters, results are likely to be susceptible to a recall bias, and no question inquired about the time between the consultation and completion of the questionnaire. The richness of the qualitative data was limited by responses being provided only in written form and therefore semistructured interviews would be recommended for future research.^33^

Further research could focus on examining experiences from both the perspective of PwIIH and the HCP and how this can affect the patient–HCPs relationship, accessibility and ultimately the health outcomes. Increasing the knowledge base for both PwIIH and HCPs regarding the role of weight in IIH is important and seen as a priority.^18^ Understanding of the actual pathways and pathogenic role of body weight and weight gain in IIH will help HCPs discuss this aspect of the disease confidently in IIH and reduce HCP weight stigmatisation.^10^ Effective communication on this challenging topic will help make PwIIH feeling more ‘heard’ and ‘accepted’.

## Conclusion

This study showed many PwIIH experience negative emotions when HCPs communicate with them about their body weight, its role in IIH and how to manage weight reduction. They call for improvements to be made to the weight management experience in the context of IIH. Further research is needed to understand the HCP perspective on body weight and weight management communication in IIH. Targeted training and ready access to weight management services are required to address weight stigma and facilitate effective communication between HCPs and PwIIH.

## Data Availability

No data are available. The data are not shareable due to the qualitative nature of data collection, in order to maintain participant anonymity.
