# Supplementary material for: Weight management communications in idiopathic intracranial hypertension: challenges and recommendations from the patients’ perspective
Source: BMJ Neurol Open. 2023 Dec 9;5(2):e000527. doi: 10.1136/bmjno-2023-000527 (PMC10729070; doi:10.1136/bmjno-2023-000527)
Supplement: Supplementary data [file bmjno-2023-000527supp001.pdf]

SUPPLEMENTARY FILE 1

Q1: Which health care professionals advised you to lose weight? Please check all that apply

- ❑ Neurologist
- ❑ Neurosurgeon
- ❑ Ophthalmologist
- ❑ Nurse
- ❑ GP

Q2: Did the person who asked you to lose weight ask your permission to discuss your weight with you?

- ❑ Yes
- ❑ No

Q3 How did the person who advised you to lose weight discuss the subject of weight with you? (Please do include quotes if you can remember the words/ language used and detail both the positives and negatives).

Q4 How did being advised to lose weight make you feel?

Q5 Have you been made to feel that IIH was your fault because of your weight?

- ❑ Yes
- ❑ No

Q6 Did you find the person who told you to lose weight empathetic and supportive?

- ❑ Yes
- ❑ No

Q7 Were you happy with how the subject of losing weight was approached with you?

- ❑ Yes
- ❑ No

Q8 Are there any words or language that you would prefer medical professionals to use if they need to discuss your weight with you?

Q9 How could the experience of being advised to lose weight have been improved?

Q10 Do you feel that you have had less favourable treatment because of the link between IIH and obesity?

- ❑ Yes
- ❑ No

Q11 When you were advised to lose weight were you offered support to do this?

- ❑ Yes
- ❑ No

Q12 If you were offered support to lose weight please detail the support that you were offered?

Q13 Was the support offered helpful and appropriate?

- ❑ Yes
- ❑ No

Q14 If you were not offered support to help lose weight did you ask for support?

- ❑ Yes
- ❑ No

Q15 What support would you like the professionals who look after you to offer to help with weight management?

Q16 If you were advised to lose weight were you given a weight loss goal?

- ❑ Yes
- ❑ No

Q17 If you were given a weight loss goal did this feel achievable for you?

- ❑ Yes
- ❑ No

Q18 Are there any other comments you would like to add on obesity stigma and IIH?
