# Supplementary material for: Weight management communications in idiopathic intracranial hypertension: challenges and recommendations from the patients’ perspective
Source: BMJ Neurol Open. 2023 Dec 9;5(2):e000527. doi: 10.1136/bmjno-2023-000527 (PMC10729070; doi:10.1136/bmjno-2023-000527)
Supplement: Supplementary data [file bmjno-2023-000527supp002.pdf]

SUPPLEMENTARY FILE 2

Illustrative quotes from PwIIH about how the experience of discussing weight management could be improved.

Categories and Sub-categories	Participant Quotations
1. Detail the relationship between IIH and weight	
1a. Provide an explanation	"An actual conversation explaining the facts on losing weight and how it will help with the condition."<br><br>"Explain more about how IIH happens and how weight loss can sometimes help."
1b. Acknowledge weight as a risk factor, not a cause	"…the condition is idiopathic so to blame it all on weight is completely unfair. There are a number of obese people that don't have it and a number of normal weight range that do. Weight loss could help, doesn't mean it will for all."<br><br>"… [weight loss] has helped some people reduce symptoms and put them into a remission but it sometimes does and doesn't work just like anything else… If it [weight] was the cause [then] every overweight female of childbearing age would have IIH so don't say

	it's the cause when it's not in fact true, there is more to the puzzle…"
1c. Provide evidence	"Maybe back up the claim with actual scientific proof (I personally have never been shown any by any professionals despite multiple requests for it)."<br><br>"There has been very little research into IIH (comparatively) and they [healthcare professionals] are very open about the fact that they don't know the direct cause."
2. Individualise care	
2a. Consider the impact of IIH symptoms	"Any support would NEED to take into account the fact that many IIH sufferers are unable to exercise due to fatigue, exhaustion and pain either caused by the condition or side effects of the condition."<br><br>"Unless you've personally struggled with weight loss while ill and feeling like cr*p most days, you have no idea of the difficulty. Simply telling someone to lose weight isn't proactive. It's like telling an athlete to run faster."
2b. Investigate co-morbidities	"I struggle with weight loss due to other conditions impacting on me but my care is in no way co-ordinated."

	"To understand that I have other health conditions that impact on my ability to lose weight and help me identify treatments that may work in that context."
2c. Explore mental health	"Every time I try to explain that the reason I struggle to lose weight is because of my mental health, the message I get is that it's my fault and I should just do it regardless. It genuinely feels like I'm talking to a brick wall."<br><br>"Taking mental health more seriously, as it has a huge effect with IIH. Overeating comes from a dark sad place… Perhaps looking at it from a more mental health support perspective (which should be the case for all people with chronic conditions)."
2d. Get to know the 'person'	"…give me the opportunity to explain about myself instead of a preconception and tell me the same thing they say to everyone that they need to lose weight not everyone is the same therefore every patient should be dealt with differently and as an individual and not all put in the same judgment…"<br><br>"These doctors might see cases off IIH all day but to every patient with IIH this is all new and

	daunting for us so the last thing we need is to be treated like a number and like we not even worth getting to know.”
3. Give advice	
3a. Avoid oversimplistic advice of ‘eat less, move more’	“The worst is “just eat healthier and exercise more”, it’s not that simple.”<br><br>“To realise that weight management is a complex thing. It is not just calories in calories out, but an emotional thing and a time management thing and an organization thing too. We can’t do it just cause you say so, or just cause we want to. If that was the case it would be done, since we wouldn’t want to deal with IIH.”
3b. Provide realistic weight loss targets	“…there also needs to be realism! If you are told lose 10kg in six months, you are being set up to fail. There needs to be smaller, more reasonable goals - and support.”<br><br>“Being realistic and discussing how much to lose or a goal that they would expect it to make a difference. Saying ‘lose weight’ is open ended and could mean anything from 2lbs - 2stone. Quantify how much weight, explain why this plays a part in improving symptoms and how to do this effectively and safely.”

4. Provide support	
4a. Discuss the available options	“Maybe offering options for weight loss support as opposed to just being told to lose weight and that being the end of the conversation.”<br><br>“Options and methods: some things work better for some people than others. Offering options helps people feel in control, and accepting that sometimes things don't work.”
4b. Organise referrals to other healthcare professionals	“Maybe offer some help from dietitian or weight management clinic instead of being bluntly told you’re overweight and then left to deal with it yourself.”<br><br>“… even if the doctor had sent a referral to things like an exercise physiologist or any other service available it would have helped. So doctor education on what is available would help.”
4c. Provide resources for self-management	“Basic support. If drs had a leaflet that had info for people to take away that would at least have made me feel less alone and overwhelmed.”<br><br>“Have resources ready and available to provide practical support.”

5. Adapt communication	
5a. Ask permission	"By asking if they could discuss my weight with me, asking if I would like advice and/or support instead of them just telling me to lose weight."<br><br>"Also ask a patient if they want to be weighed. If it isn't necessary for the appointment or medication dosing it should be optional."
5b. Show empathy	"More empathy, understand the fact that for some losing weight is not a simple solution and that most of us didn't chose to be fat."<br><br>"Show empathy and help to find a way forward together."
5c. Use people-first language	"Stop saying the word obese every other word when talking to me… I don't like the word fat or obese."<br><br>"I wish they'd ditch the "morbidly" or "extremely" as it's dehumanising."
5d. Avoid using shaming language	"Use words that don't shame the patient or blame you for causing your illness."<br><br>"Be understanding and speak to me like I'm a human, fat shaming is not helping anybody.

	People forget we are human too. We don't choose to go blind. We need help."
5e. Engage in a two-way dialogue	"I think we need to have our voices heard more. I always feel like I'm being talked at and when I speak or have opinions I feel they were quickly dismissed."<br><br>"Explore my attitudes and motivations to my weight and weight loss. Understand and acknowledge my current efforts and provide guidance as to how we can make a plan TOGETHER to make this more successful."
5f. Ensure privacy	"Keeping conversations about my weight to private rooms rather than the ward where other people can hear."<br><br>"I've had nurses come up to me in a full waiting room to say out loud I need to weigh you and take me off elsewhere. No one else seeing other neuros were weighed- was embarrassing."
